# Multimodal Stimulation in a Microfluidic Device Facilitates Studies of Interneurons in Sensory Integration in *C. elegans*

**DOI:** 10.1002/smll.201905852

**Published:** 2020-01-31

**Authors:** Yongmin Cho, Sol Ah Lee, Yee Lian Chew, Kirby Broderick, William R. Schafer, Hang Lu

**Affiliations:** ^1^ School of Chemical & Biomolecular Engineering Georgia Institute of Technology Atlanta GA 30332 USA; ^2^ Neurobiology Division Medical Research Council Laboratory of Molecular Biology Cambridge CB2 0QH UK; ^3^ Present address: Department of Systems Biology Harvard Medical School Boston MA 02115 USA; ^4^ Present address: Molecular Horizons and School of Chemistry and Molecular Bioscience University of Wollongong & Illawarra Health and Medical Research Institute Wollongong NSW 2522 Australia

**Keywords:** *C. elegans*, microfluidics, multisensory integration, PVC interneurons

## Abstract

Animals' perception and behavior involve integration of multiple sensory modalities. *Caenorhabditis elegans* is a useful model for studying multimodal sensory integration, as it has well‐characterized neuronal circuits in a relatively simple nervous system. However, most studies based on functional imaging have only been conducted on single modal stimuli, because well‐controlled multimodal experiments for *C. elegans* are technically difficult. For instance, no single systems currently deliver precise stimuli with spatial, temporal, and intensity control, despite prior hypotheses that interneurons do integrate these sensory inputs to control behavior. Here, a microfluidic platform that can easily deliver spatially and temporally controlled combination stimuli to *C. elegans* is presented. With this platform, both sensory and interneuron activity is measured in response to mechanical and chemical stimulations in a quantitative and high‐throughput manner. It is found that the activity of command interneuron PVC can be modulated by prior stimulation both within the same and across different modalities. The roles of monoaminergic and peptidergic signaling are further examined on the process of multimodal integration through PVC activity. The approach exemplified here is envisioned to be broadly applicable in different contexts to elucidate underlying mechanisms and identify genes affecting multisensory integration.

## Graphical abstract

Here, a microfluidic platform that can easily deliver spatially and temporally controlled combination stimuli to *Caenorhabditis elegans* is presented. Both sensory and interneuron activity is measured in response to mechanical and chemical stimulations in a quantitative and high‐throughput manner, and it is found that activities of an interneuron can be modulated by prior stimulation both within the same and across different modalities.

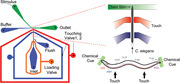

## 1 Introduction

For all animals, perception and behavior are usually multisensory processes, often involving stimuli such as taste, smell, and touch. Each of these cues are detected by distinct sensory neurons highly specialized for a specific input. Integrating sensorycues from multiple modalities allowsanimals to better extract relevant information from a complex environment to facilitate decision‐making.^1^, ^2^, ^3^ Across the animal kingdom, multicue stimuli often elicit enhanced responses compared to single‐cue events.^4^, ^5^, ^6^ The enhancement is often a result of integration of information from multiple modalities. For example, sensitization is a nonassociative learning process in which repeated administration of a stimulus results in an enhanced response.^7^ In comparison, habituation is another form of nonassociative learning in which an animal decreases or ceases responding to a stimulus after repeated or prolonged stimulation.^8^ Further, in some situations, a sensitizing stimulus can override the effects of habituation, a phenomenon called dishabituation.^9^, ^10^, ^11^, ^12^ Together, these complex phenomena provide crucial mechanisms that allow animals to rapidly adapt to a changing environment by adjusting sensory responsiveness. Although these phenomena have been described, it is not completely known how neurons reliably process and integrate sensory signals across modalities in order to produce precise and accurate responses.

*Caenorhabditis elegans*, a microscopic soil nematode with 302 neurons and well‐defined synaptic connectivity, is a complex and yet tractable system to address the neuronal basis of multisensory integration.^13^ Classically, the nervous system is composed of three types of neurons: sensory neurons that interface directly with the environment, interneurons that integrate the sensory and internal signals, and motor neurons that convey the commands to muscles. It is known that *C. elegans* can respond to a variety of sensory cues, such as smell, taste, touch, oxygen level, and temperature.^14^, ^15^, ^16^, ^17^ These sensations allow the animals to find food and gauge the extent of danger in their environment, and then modulate their behavior accordingly.^18^ The sensory components for each modality have been largely mapped, in part with the guidance of a complete wiring diagram.^19^, ^20^ For example, six touch receptor neurons (ALML/R, PLML/R, AVM, and PVM) detect gentle mechanical stimuli, and the nociceptive neurons ASH and PHB (which are also known to sense chemical repellents) synapse onto four shared pairs of command interneurons with the touch receptors.^21^, ^22^ The command neurons are connected to the motor circuit responsible for modulating forward and backward locomotion, leading to the appropriate behavioral outputs in response to the stimuli.^14^ Although the anatomical connectivity of these sensory systems and the role of each sensory neurons have been independently characterized, the integration of these signals and whether and how the neurons modulate each other's activities are largely unknown. In particular, the cellular and molecular mechanisms are not well‐defined. This is mainly due to the limitations of the current tools for physiological and functional imaging assays; without using microfluidics, one would have to resort to simultaneously delivering mechanical stimulus using micromanipulators and chemical stimulus using microspritzer on glued worms.^23^, ^24^, ^25^, ^26^

Microfluidics has been widely used to address some fundamental limitations in experiments on small biological specimen, including *C. elegans*.^27^ Due to the match in length scales and the ability to manipulate laminar flow, it has been shown that microfluidics can precisely handle microsized samples and control experimental conditions, and often with much lower reagent consumption. Recent studies further demonstrate the advantages of microfluidics for automated and high‐throughput experiments when coupled with software and additional hardware.^28^, ^29^, ^30^, ^31^ Further, microfluidics can be designed to work together with any modality of optical microscopy, allowing for the imaging of fluorescent markers such as calcium indicators.^32^

While microfluidic platforms have been widely developed for *C. elegans* to monitor neuronal activity and behavior under spatially or temporally controlled stimuli, including chemical, mechanical, oxygen, or temperature gradients,^32^, ^33^, ^34^, ^35^, ^36^, ^37^, ^38^ simultaneously recording neuronal activity under well‐controlled multimodal stimulation via for instance, calcium imaging, has not yet been shown. This is mainly due to the complexity of these experiments and the demands and constraints each experimental component places onto the integrated assay system. Pneumatic controls for flow and actuations on different parts of the microfluidic system can often place conflicting demands; for instances, the device would need to maintain delicate pressure balance when delivering either mechanical or chemical stimulus while maintaining the worms in the focal plane.

**Figure 1:**
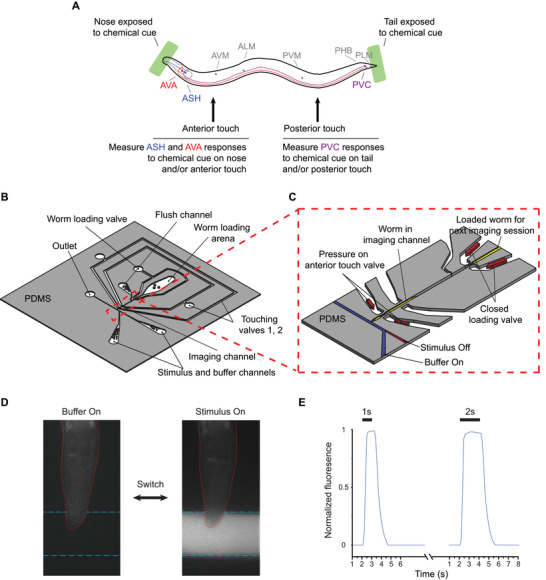
Design of microfluidic system for delivering both chemical and mechanical stimuli to *C. elegans*. A) Schematic of a multisensory assay for presenting stimuli and target neurons (colored) in this study. B) Overview of device design (detail in Figure S1 in the Supporting Information). C) Zoomed‐in image for imaging channel showing mechanical and chemical stimulation modules. Worms can be sequentially loaded and unloaded by controlling the pressure in a worm loading valve and a flushing channel. D) Example images of worm in the device are exposed to buffer and stimulus, mixed with a fluorescein dye. E) Dye visualization experiments using a fluorescein dye to simulate the stimulus with different durations (1 s: *n* = 5 and 2 s: *n* = 7).

A further challenge is the spatial and intensity control of stimuli. In *C. elegans* nervous system, each sensory neuron has different receptive fields and response thresholds, all in a minute specimen (diameter of tens of micrometers). Among the six gentle touch neurons, AVM and ALMR/L neurons can sense and transduce weak mechanical stimuli on the anterior region, while PLMR/L neurons sense in the posterior (**Figure** **1**A); PVD, a harsh touch neuron, can sense relatively stronger mechanical stimuli than these gentle touch neurons. Interneurons such as PVC are directly connected to not only both the gentle and harsh touch neurons but also chemosensory neurons (e.g., PHB, located in the tail). Further, most chemical stimulation is delivered via flow, which could potentially activate some mechanosensory cells. Therefore, in order to understand sensory integration in interneurons, the experimental system has to deliver stimuli to be at specific locations, with the intensity and duration intended, and have these cues to be independently controlled and modulated. The complexity is perhaps why such integrated systems have not been attempted prior.

Here, we present a microfluidic device that addresses these challenges. Our system can deliver simultaneous mechanical and chemical stimuli to *C. elegans* with precise spatiotemporal and intensity patterns, while recording single‐cell neuronal responses via calcium imaging. Specifically, we integrated deformable polydimethylsiloxane (PDMS) membranes that can deliver well‐controlled mechanical stimuli^39^, ^40^ with a module for the controlled delivery of chemical stimuli via off‐chip solenoid valves. We demonstrate that both sensory neurons and interneurons can be activated by distinct types of stimulation. We used this system to examine the propagation of sensory information in a neural circuit by monitoring sensory and interneuron responses to combinations of controlled mechanical and chemical stimuli. We investigate how a prior stimulus can modulate the activity of the command interneuron PVC, and report for the first time that PVC activity can be sensitized in the same or across different modalities. Finally, we tested genetic mutants to assess the contribution of neuromodulators for the PVC sensory integration phenotype.

## 2 Results and Discussion

### 2.1 Integrated Microfluidic System for the Automated Precision Delivery of both Mechanical and Chemical Stimuli

We developed a monolithic microfluidic platform in which we can deliver chemical and mechanical stimulations to specific spatial locations of a worm body (Figure 1B,C; Figure S1, Supporting Information; see the Experimental Section for details). This device integrates PDMS actuators for the delivery of mechanical stimulus,^39^, ^40^ with a chemical delivery module. Using the platform, we can touch either the anterior or posterior regions of the worm body and deliver chemical stimuli to either the head or tail of the worm (Figure 1A). Since sensory neurons in *C. elegans* have a specific receptive field, we designed our platform to selectively activate the neurons with spatial resolution. For a functional imaging experiment, individual animals can be loaded into an imaging channel, and depending on the experimental purpose, mechanical stimuli can be delivered through two pairs of in‐plane PDMS membrane structures targeting the anterior/posterior half of the animal, and/or chemical stimuli can be delivered and controlled by off‐chip solenoid valves (Figure 1B,C).

The PDMS membrane structures are pneumatically actuated, and when deflected, exert a mechanical stimulus over animals trapped in the imaging channel. The duration and intensity of stimuli can be readily controlled, allowing for various patterns of stimulation application, such as testing for a graded response, habituation, dishabituation, and sensitization. One additional actuated structure acts as a worm loading valve, and a flush channel allows for the loading of individual worms sequentially (Figure 1B; Figure S1, Supporting Information). The worm loading valve and flush channel, PDMS actuators, and chemical stimulus module are all connected to a pressure source via individually controlled off‐chip solenoid valves that are computer controlled, and thus completely automated, allowing for high‐throughput experimentation. Another important feature of our platform is that the optimized channel sizes (i.e., 50 µm × 50 µm channel, matching the width and height for day 1 to day 2 adult worms) and a three‐step vertical tapering of the imaging channel minimize the movement of head or tail part of worms (Figure S1C, Supporting Information). This feature is important for high‐quality functional imaging without the need for other immobilization techniques (e.g., chemical anesthetics) that may affect neuronal activity. By confining the head or tail of the animal in the vertical direction, neurons of interests remain largely in plane for the duration of the experiment, and the system thus delivers high‐quality imaging data with less motion artifact overall.

While systems to deliver chemical stimuli exist, e.g., Chronis et al.'s four‐flow system,^36^ in practice, these designs require the pressure to be delicately balanced while moving streamlines laterally in the chip. Instead of using a four‐flow system for the chemical stimulus delivery, we choose off‐chip solenoid valves. Our implementation is simpler and removes the complication of combining many flow and control channels and thus more robust during operation.

To characterize the temporal dynamics of chemical stimuli in our chip, we quantified the fluorescence intensity profile near the animals' nose in a mock experiment using a fluorescein dye in the stimulus stream (Figure 1D,E). Figure 1E shows that the system has a rise time (defined as time to reach 90% of max intensity) of 0.6 ± 0.1 s and a fall time of 1.2 ± 0.1 s. The mixing is likely a result of the Taylor dispersion in the off‐chip tubing system, which may be further reduced by using thinner diameter or other means,^41^ and possibly the capacitance in the system (where we estimated the RC time constant to be in the range of hundreds of milliseconds), which can be further mitigated by using stiffer tubing. In experiments with varying duration of the stimuli, it is clear that the ramp‐up/ramp‐down times are independent of the durations of stimuli. In addition, our platform can deliver two different types of chemical stimuli in a highly controlled manner as a single chemical stimulus (Figure S2, Supporting Information). Using a Y‐shaped connector that can connect two chemical reservoirs and an additional off‐chip solenoid valve, we can quickly switch from the first chemical to buffer and then to the second chemical. This demonstrates that our system can reliably deliver sequences of pulses of chemical stimulus for most applications, including those to probe chemosensory responses in *C. elegans*.

When both modalities are combined during operation, the pressure control needs to be precise to not introduce undesired flow and perturbations. In our system, the pressures of the inlets, outlets, and pneumatic actuators are precisely controlled from constant pressure sources. It ensures that flow is in the designed direction with the desired magnitude and that pneumatic actuations do not induce significant change in resistance and flow aberration. Because we use pneumatic controls, both the chemical and the mechanical stimuli have high temporal precision, and can be delivered in arbitrary patterns. These robust operations thus allow the studies on how the interneurons integrate the different signals from each sensory input, as well as how prior experience modulates perception.

### 2.2 Robust Stimulation to Elicit Responses in both Sensory Neurons and Interneurons

**Figure 2:**
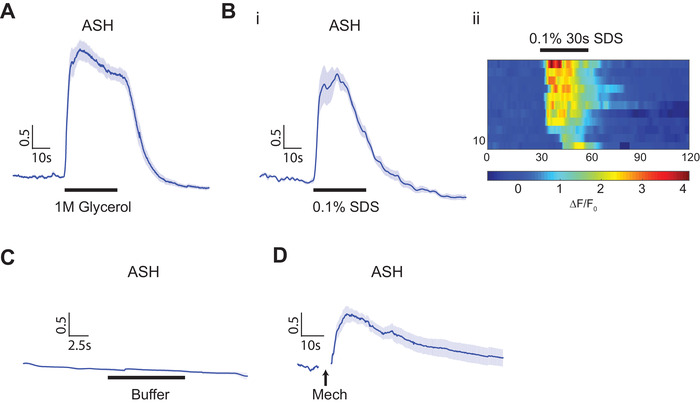
Polymodal sensory neuron, ASH, shows responses to both chemical and mechanical stimulations. A,B) Calcium transients show that ASH responses to chemical stimuli. A) 1 m glycerol (*n* = 13) and B) 0.1% SDS (i: average traces, ii: heatmap of individual traces, *n* = 11). Traces are ordered separately for each trial according to its activity at the point of stimulus being turned on. C) ASH responses to buffer‐to‐buffer changes (*n* = 10). D) ASH responses to 5 s mechanical stimulation in the anterior region (*n* = 13). Error bars represent SEM.

To demonstrate the controllability of individual stimulus modules, we monitored the activity of the polymodal sensory neuron ASH, using a genetically encoded calcium indicator, GCaMP, in response to either chemical or mechanical stimulation.^42^, ^43^ Previous studies showed that ASH responds to noxious stimuli, including solutions of high osmolality, chemical repellents, and nose touch.^22^, ^44^ We chose ASH because its responses have been characterized in many contexts, and a well‐designed system would be able to recapitulate the ASH responses quantitatively. As shown in **Figure** **2**A,B, we observed robust calcium transients in ASH stimulated by hyperosmotic solution (1 m glycerol) and detergent (0.1% SDS), with the kinetics and magnitude of responses similar to those in other platforms.^23^ In addition, we can deliver repeated chemical stimulations precisely, and ASH neurons show robust habituation to both hyperosmotic solutions and detergent as expected (Figure S3, Supporting Information). When delivering buffer‐to‐buffer exchange as a control, we saw no ASH response, which indicates that the pressure surge or shear associated with flow and flow switching are at subthreshold level in our system (Figure 2C). In contrast, when mechanical stimuli (5 s duration) was delivered using the anterior touch membrane, ASH neurons showed robust responses to the mechanical stimuli specifically as expected (Figure 2D). We thus believe that the system can deliver decoupled chemical and mechanical inputs separately and in a controlled manner.

**Figure 3:**
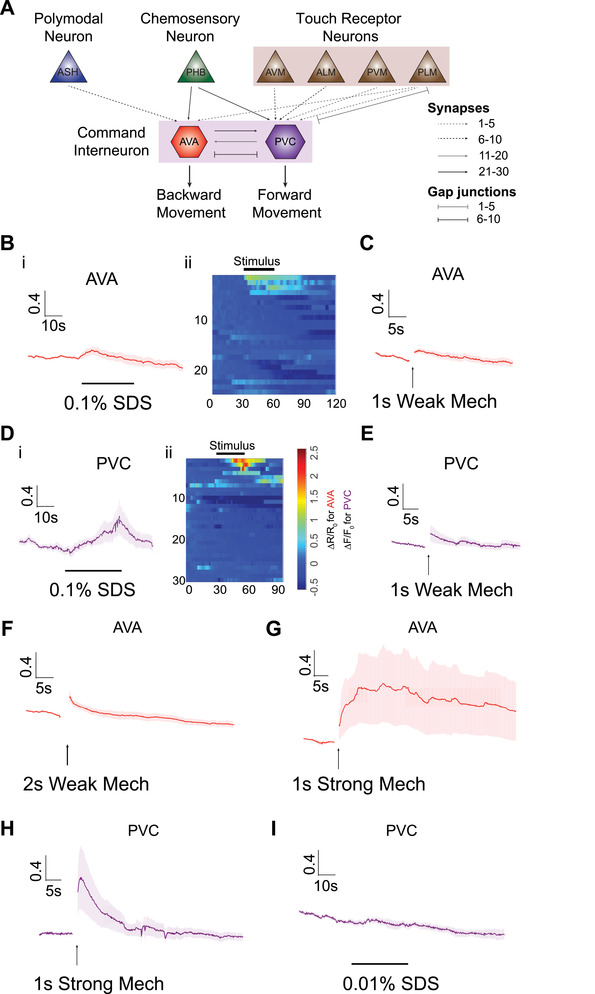
Interneurons can be activated by both chemical and mechanical stimuli in the device, and the response is graded. A) Simplified circuit diagram showing multiple sensory neurons are connected to command interneurons that mediate either forward or backward movement. B–I) Interneuron calcium responses to various conditions (AVA: chemical stimulus on nose and mechanical stimulus on anterior region, PVC: chemical stimulus on tail and mechanical stimulus on posterior region). 30 s 0.1% SDS stimulus: B) AVA (i: average traces, ii: heatmap of individual traces, *n* = 24) and D) PVC (i: average traces, ii: heatmap of individual traces, *n* = 30). 1 s weak mechanical stimulus at 20 psi: C) AVA (*n* = 14) and E) PVC (*n* = 22). Longer (2 s) weak mechanical stimulus at 20 psi: F) AVA (*n* = 12). 1 s strong mechanical stimulus at 45 psi: G) AVA (*n* = 12). H) PVC (*n* = 12). 5) Dilute (0.01%) SDS stimulus: I) PVC (*n* = 11). Error bars represent SEM.

We next turn to imaging interneurons. The *C. elegans* wiring diagram predicts direct synaptic connections between mechanical and chemical sensory neurons to command interneurons; among them are two important interneurons, AVA in the amphid, and PVC in the posterior ganglion (**Figure** **3**A).^13^ Many sensory neurons, both in the head and the tail, connect to AVA and PVC. Notably, posterior chemosensory neurons PHBL/R, which have been shown to respond to 0.1% SDS stimulus^21^ connect to both AVA and PVC. ASH and the mechanoreceptor neuron PLM are directly wired to AVA. PVC interneurons are also postsynaptic to all mechanoreceptor neurons. This wiring diagram suggests that AVA and PVC are likely to play important roles in sensory integration that drives the backward and forward movement of the animal.

In order to quantitatively assess the functional role of interneurons in sensory integration, we first asked whether these two command interneurons can be activated by controlled individual chemical or mechanical stimuli in our platform. Interneuron responses have previously been shown to be more stochastic from trial to trial, compared to sensory neuronal activity.^45^ The data on our chip recapitulated this finding. In contrast to ASH sensory neuron, which responds to chemical stimulus with a sharp rise in calcium (as shown in Figure 2A,B), AVA and PVC interneurons respond to either mechanical or chemical stimuli with a smaller magnitude and more slowly than the ASH neuron (Figure 3). In addition, the responses are more variable than those of sensory neurons. For example, if the criterion of calcium trace rising above 0.5 of Δ*F*/*F*_0_ is used to define a response, ASH neurons show a 100% response rate to 30 s 0.1% SDS stimulus perfused over the nose (average of maximum peak of Δ*F*/*F*_0_ is 2.61) (Figure 2B); in comparison, by the same definition, only 13% of animals show responses in AVA interneurons (Figure 3B), and the average response peaks at a Δ*R*/*R*_0_ of 0.21. Similarly, with 0.1% SDS simulation to the tail, only 17% of animals show responses in PVC interneurons, with an average peak of Δ*F*/*F*_0_ at 0.32 (Figure 3D).

Interestingly, both AVA and PVC interneurons show graded responses to stimuli, similar to sensory neurons. For example, when a weak mechanical stimulus is delivered (either short duration or weak intensity of stimulus), interneurons show a low probability of response (Figure 3C,E). However, when a longer duration or strong mechanical stimulation was delivered, a higher fraction of worms shows discernible responses (Figure 3F–H). For chemical stimuli, PVC shows more reliable responses to a higher concentration of SDS (0.1%), as compared to lower response fraction with lower concentration of SDS (0.01%) (Figure 3D,I). As a control, we confirmed that PVC interneurons do not show any response to buffer‐to‐buffer changes, which is not surprising as the sensory neurons (e.g., ASH) are not stimulated on our chip during buffer‐to‐buffer switch (Figure S4, Supporting Information). Generally, we observed that PVC shows more reliable responses to both single chemical and mechanical stimuli than AVA neurons given the same sensory input. This is consistent with the number of anatomical inputs from the sensory neurons onto these interneurons:^13^ while PVC receives input from PHB chemosensory neurons via 22 synapses, AVA receives input from ASH sensory neuron through only 7 synapses. In addition, PVC is directly connected to six gentle touch neurons through either chemical synapse or gap junctions; AVA is only indirectly connected to those touch neurons, except PLML/R.

### 2.3 Interneuron Integration of Sensory Information

**Figure 4:**
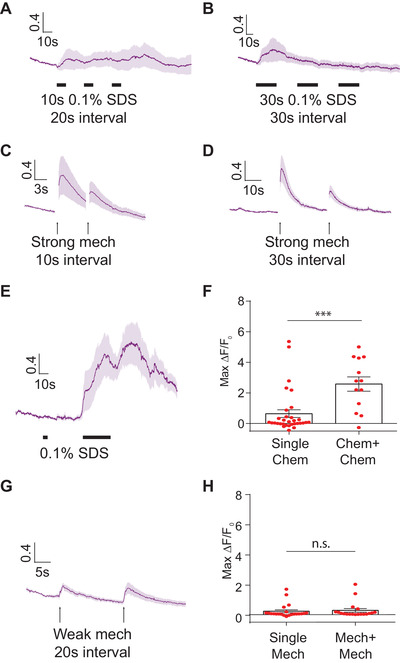
PVC interneuron responds to repeated chemical or mechanical stimuli. The activity of PVC interneurons can be differently modulated depending on the timing, intensity, and duration of stimuli. A,B) PVC responses to repeated chemical stimulations with different durations of stimuli. A) 20 s interstimulus interval with 10 s 0.1% SDS stimulation (*n* = 10) and B) 30 s interstimulus interval with 30 s 0.1% SDS stimulation (*n* = 14). C,D) PVC responses to repeated strong 1 s mechanical stimulations with different durations of interval. C) 10 s interstimulus interval (*n* = 21) and D) 30 s interstimulus interval (*n* = 16). E) Applying a subthreshold SDS stimulus (5 s 0.1%) enhances the PVC responses to a subsequent 30 s 0.1% SDS stimulus (*n* = 13). F) Quantitative comparison of the maximum responses of the PVC calcium transients, with and without the initial subthreshold stimulus (single chem: *n* = 30, chem + chem: *n* = 13, Mann–Whitney test, ***p < 0.001). G) 20 s interstimulus interval with 1 s weak mechanical stimuli (*n* = 19). H) Quantitative comparison using the maximum responses of calcium transients from (G) (single mech: *n* = 22, mech + mech: *n* = 19). F,H) Data points represent maximum responses of each trial, using single chemical and mechanical data from Figure 3D,E, respectively.

We next asked how the interneurons might integrate different modalities of sensory inputs. Previous work has shown that presenting repeated mechanical stimuli can cause habituation in activities of mechanosensory neurons and behavioral outputs.^41^, ^43^, ^44^ In contrast, prolonged vibration sensitizes the touch receptor neurons.^46^ To examine under what conditions interneurons can be sensitized or habituated, we delivered repeated stimuli to animals at different intensities and at various durations and intervals (**Figure** **4**). With chemical cues (0.1% SDS), repeated shorter stimuli (10 s) can produce a sustained response in PVC (Figure 4A). In contrast, when using longer repeated chemical stimulation (30 s duration per stimulus), the response magnitude was reduced for subsequent stimulus exposure (Figure 4B), suggesting habituation is taking place. Similarly, strong mechanical stimuli in the posterior region of worms induce habituated responses in PVC for both short and long interstimulus interval (Figure 4C,D). These results are consistent with previous observations that habituation is dependent on stimulus strength and interstimulus durations.^47^, ^48^

Interestingly, the activity of PVC to subthreshold chemical stimuli can considerably enhance responses to subsequent chemical stimulation: when worms are initially exposed to subthreshold chemical stimuli (5 s duration), PVC shows significantly higher calcium responses to 30 s 0.1% SDS (Figure 4E), compared with animals only exposed to 30 s 0.1% SDS (Figure 3D). Figure 4F shows the summary statistics of the two experimental conditions, demonstrating that it is in a fraction of the animals that these sensitization effects are occurring. In contrast, under a wide variety of conditions we tested, we did not observe clear sensitization effect in mechanosensation (as exemplified in Figure 4G,H). Together, these data suggest that the timing, intensity, and duration of stimuli are critical for sensitization or habituation. While both interneurons and sensory neurons can distinguish the intensity of the stimulation and can habituate, different from sensory neurons, interneurons can be sensitized in some scenarios.

### 2.4 Multimodal Sensory Integration on PVC Interneuron

**Figure 5:**
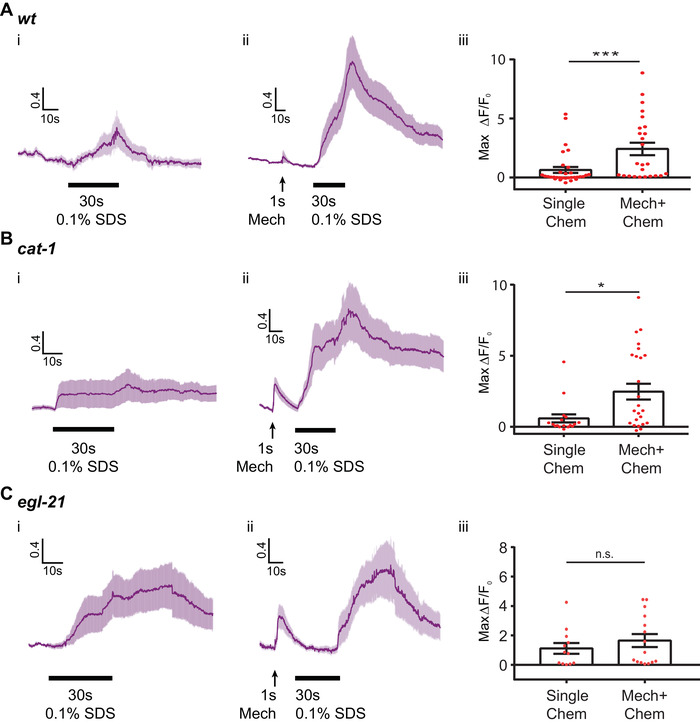
Activity of PVC interneurons responding to chemical stimuli can be modulated by prior mechanical stimulus. This effect likely involves peptidergic signaling. A–C) Wild‐type, *cat‐1* and *egl‐21* mutants were tested for PVC multisensory integration under the following conditions: i) single 30 s 0.1% SDS stimulus on tail, ii) 1 s weak mechanical stimulus on posterior region and then 30 s 0.1% SDS stimulus on tail, and iii) quantitative comparison using the maximum responses of calcium transients (Mann–Whitney test, *p < 0.05, ***p < 0.001). Data points represent maximum responses from individual trials. A) wild‐type (single chem: *n* = 30 and mech + chem: *n* = 25), B) *cat‐1* (*e1111*) mutant (single chem: *n* = 17 and mech + chem: *n* = 26), and C) *egl‐21* (*n476*) mutant (single chem: *n* = 12 and mech + chem: *n* = 15). Error bars represent SEM. Single stimulus experiments for wild‐type are the same as in Figure 3D.

Next, since we observed sensitized activity of PVC neurons in chemosensation, we asked whether and how PVC interneurons integrate input signals from cross‐modal stimuli. As baseline cases, we tested the single stimulus in either modality (i.e., 30 s 0.1% SDS stimulation to the tail, or 1 s weak mechanical stimulation to the posterior region). PVC shows a moderate response with a low probability of response (≈20%) (**Figure** **5**A‐i; Figure S5A‐i, Supporting Information). In contrast, when animals are exposed to cross‐modal subthreshold stimuli (i.e., chemical before mechanical stimulus, or vice versa), the magnitude of the PVC response is greatly enhanced, with a larger probability of response (Figure 5A; Figures S5A and S6, Supporting Information). Specifically, we found that animals pre‐exposed to 1 s weak mechanical stimulus demonstrated a significantly higher PVC calcium response to SDS, compared with animals exposed to SDS alone (Figure 5A‐ii; Movie S1, Supporting Information). Conversely, pre‐exposing the animal to a single short chemical stimulus to the tail can also sensitize the PVC responses to a weak mechanical stimulus (Figure S5A‐ii and Movie S2, Supporting Information). This cross‐modal modulation of neuronal activity is seen both in terms of the magnitude of the individual responses and the fraction of responding animals (Figure 5A‐iii; Figures S5A‐iii and S6, Supporting Information). These results indicate that both weak mechanosensory and weak chemosensory stimulations can increase the excitability of PVC interneurons to a second stimulus, independent of the exact nature of the stimulus. In contrast, AVA did not show this type of phenotype under our experimental conditions (Figure S7A–C, Supporting Information).

### 2.5 Effects of Neuromodulators on PVC Cross‐Modal Sensitization

Previous studies have implicated both monoamine and peptide neuromodulators in driving sensitization in many species.^49^, ^50^, ^51^ Thus, we hypothesized that neuromodulators may be required for the information processing in cross‐modal integration in PVC. We first asked whether mutants lacking monoamine neurotransmitters display the PVC cross‐modal integration phenotype. *cat‐1* encodes a synaptic vesicular monoamine transporter (e.g., dopamine and serotonin).^52^, ^53^, ^54^ Similar to wild‐type PVC activity, *cat‐1(e1111)* mutants show a lower magnitude and less reliable responses to either single chemical (Figure 5B‐i) or single mechanical stimulations (Figure S5B‐i, Supporting Information). When *cat‐1* mutant animals are exposed to subthreshold mechanical stimulus first and then exposed to a second chemical stimulus, PVC neurons still show enhanced responses compared to single modal stimulation (Figure 5B‐ii). However, the enhancement (as compared to single chemical stimulus) is less reliable compared to the wild‐type case. (Figure 5B‐iii); if the criterion of area‐under‐the‐curve of calcium transients is used as a parameter instead of the maximum peak value, *cat‐1* mutant animals do not show the statistically significant sensitized phenotype (Figure S8B, Supporting Information). Priming the response to mechanical stimulus with an initial chemical stimulus also produces a reduced sensitization effect in *cat‐1* mutants, compared to wild‐type (Figure S5B, Supporting Information). These experiments suggest that monoamines play a minor but noticeable role in sensitization.

We next tested whether neuropeptide signaling also plays a role in PVC cross‐modal integration. *egl‐21* is required for processing of FMRFamide‐like and neuropeptide‐like peptides.^55^ We found that there are moderate defects in *egl‐21* mutants: when pre‐exposed to mechanical stimuli, *egl‐21(n476)* mutants show a similar response, as compared to that of single chemical stimuli alone in the maximum Δ*F*/*F*_0_ comparison (Figure 5C); the area‐under‐the‐curve of calcium transient comparison (Figure S8C, Supporting Information) also demonstrates a defect when compared to wild‐type. We note that similar to *cat‐1*, the responses of *egl‐21* mutants to mechanical sensitization of chemical responses do not show statistical significance compared to single mechanical stimulus (Figure S5C, Supporting Information). Interestingly, besides the defect in magnitude of sensitization, *egl‐21* mutants also display a delay in the production of PVC calcium transients in response to chemical stimuli with mechanical pre‐exposure, which is robust and statistically significant (Figure 5C; Figure S9, Supporting Information); this is not observed in either wild‐type or *cat‐1* mutants. Together, these results suggest that FMRFamide‐like peptides and neuropeptide‐like peptides in general may be partially responsible for facilitating cross‐modal integration in PVC neurons under our experimental conditions. These observations could not have been possible without the well‐controlled multimodal device.

## 3 Conclusion

Fundamental studies of multimodal sensory integration require experimental platforms that can deliver spatiotemporally controlled stimulations and allow quantitative functional imaging in multiple modalities. For *C. elegans*, although many exquisitely controlled conventional or microfluidic systems for sensory stimulation exist, most of them are dedicated to single modalities. The lack of instrumentation currently bottlenecks the study of multimodal sensory integration. In this study, we demonstrated a microfluidic device that allows for the delivery of controlled mechanical and chemical stimulations to well defined regions of *C. elegans* body, while simultaneously allowing the optical recording of neuronal responses. We also automated the entire operation of the platform, thus facilitating precise temporal control of the cues, increasing throughput, and minimizing potential error and user bias.

Using this system, we can elicit and image responses in both sensory neurons and command interneurons in anterior and posterior parts of the body to both chemical and mechanical stimuli. We found that both sensory and interneurons can show dose‐dependent responses conditioned to the strength and duration of stimulations. More importantly, we showed here that the activity of the command interneuron PVC can be modulated by prior sensory inputs and that this modulation can occur in both same‐ and cross‐modal stimulus patterns. This observation may point to an interesting ecologically relevant strategy for animal behavior that the reliability of the escape response depends both on the stimulus and on the current state of circuit activity, as influenced by experience. Further, we tested the effect of genes that regulate neurotransmitter and neuropeptide signaling, and found that peptidergic signaling mediates the timing of interneurons' sensitized responses. The functions of neuropeptides in multisensory integration as well as in learning and memory thus may allow animals to create context‐dependent adaptations at both the neuronal and behavioral levels.

We envision that our microfluidic system's capability can greatly expand the repertoire of functional imaging assay conditions to allow the dissection of neural mechanisms underlying multisensory integration and the involvement of neuromodulators. First, our platform can be easily adapted for the delivery of more complex (spatial and temporal) patterns of chemical and mechanical stimuli. For example, one can activate other target neurons by relocating the position of PDMS actuator for nose touch instead of anterior touch. Moreover, various temporal patterns of stimulation can be tested in our platform (e.g., simultaneous multicue stimulation; Figure S7D–F, Supporting Information) to study neural circuit motifs that regulate multisensory behavior. Second, the platform allows for imaging motor neuron and muscle activity to examine how information processing at the sensory and interneurons can be translated to motor neurons and muscles, and how sensorimotor response under multimodal condition is generated. Lastly, while we showed how this system is used to study isolated neurons in a circuit during multimodal behavior, the system can be easily applied to other imaging systems, such as for whole‐brain imaging, since the microfluidic device and the auxiliary setup do not impose constraints on the optical setup. With our platform, it is now possible to study how neurons of *C. elegans* encode external cues and how information is processed at the levels of single cells, neural circuits, and whole brain.

## 4 Experimental Section

##### Device Design and Fabrication

The device consisted of a worm inlet/outlet, flush channel, imaging channel (50 × 50 µm wide and high for day 1 to day 2 adults), two inlets for stimulus delivery, and three sets of actuated PDMS membranes. The width of actuated PDMS membrane was 150 µm, and the distance between first and second sets of membranes was 200 µm and second and third sets of membranes was 250 µm (Figure S1, Supporting Information). Since worms were not immobilized using drugs, animals' heads or tails could move in the imaging channel of the microfluidic chip. This movement sometimes blurs images. To reduce the movement of head or tail part of the worms, a three‐step vertical tapering of the imaging channel was used to restrict the out‐of‐plane movement. The thickness of first layer was 20 µm and second and third layers were 15 µm for the 50 µm deep imaging channel; these layers were created by SU‐8 2015 negative photoresist (MicroChem) using standard photolithographic techniques.^56^, ^57^

To create an easily actuated PDMS structure to touch worms, 23:1 PDMS mixture (Sylgard 184, Dow Corning, USA) was deposited via spin coating to create a thin layer (speed: 1000 rpm, ramp: 5 s, and spin time: 30 s). For the top layer, a 10:1 PDMS mixture was directly poured onto a featureless master to create a thick and mechanically rigid handle layer. Both layers were then placed into an 80 °C oven for 25–30 min until the control layer PDMS was rigid but sticky. After that they were manually aligned, additional 10:1 PDMS was poured on top of the layers to fill the gap between the aligned layers and the Petri dish, and then the Petri dish was placed into an 80 °C oven for overnight to create a rigid handling layer for the device.

After curing, PDMS devices were cut to size, and featured PDMS chunks were created by puncturing the cured PDMS with sharpened gauge needles (19 gauges for three PDMS actuators and 18 gauges for others). The punched holes were cleaned with air‐gun and feature side of devices was then cleaned with scotch tape. The prepared devices were exposed to air plasma for 25 s before being placed and covalently bonded to a glass surface to create closed channels. Right after this bonding procedure, the devices were placed on top of a 150 °C hot plate for 3 min to increase the adhesion between PDMS and the cover glass.

##### *C. elegans* Strains and Maintenance

*C. elegans* were maintained on nematode growth medium (NGM) plates seeded with *Escherichia coli* strain OP50 under standard conditions.^58^ Hermaphrodites were used for all experiments. The following strains were used in this study: CX10979 *kyEx2865[Psra‐6::GCaMP3, Pofm‐1::GFP]*, ZM9059 *hpIs580[Prig‐3::GCAMP6::mCherry]*, QW625 *zfIs42[rig‐3p::GCaMP3::SL2::mCherry + lin‐15]*, GT243 *aEx2[Pglr‐1::GCaMP6(s); punc‐122::GFP]*, AQ4298 *egl‐21(n476); aEx2[Pglr‐1::GCaMP6; Punc‐122::GFP]*, and AQ4300 *cat‐1(e1111); aEx2[Pglr‐1::GCaMP6; Punc‐122::GFP]*.

A hatch‐off procedure was used to synchronize worms. Briefly, adult hermaphrodites laid eggs overnight at 20 °C on NGM plates. Hatched larvae and adult were removed from the plates with three successive washes with M9 buffer. The embryos remaining on the plate were incubated at 20 °C for only 1 h. Highly synchronized L1 larvae that hatched in the 1 h window were washed off and transferred to new NGM plates. Ethics approval was not required for the experiments using animals in this study. The Institutional Biosafety Committee's approved protocols for biosafety were followed.

##### Calcium Imaging and Data Analysis

All imaging experiments were performed on a Leica DMIRB inverted microscope using a 40× air objective (N.A. 0.75). Video sequences were captured using a Hamamatsu EM‐CCD camera with 100 ms exposure time. Simultaneous two‐color imaging was performed using a DV2 beamsplitter (Photometrics) containing a green fluorescent protein (GFP)/red fluorescent protein (RFP) filter set. Excitation light for fluorescent imaging was delivered through a previously developed projector system.^59^

Before calcium imaging, it was necessary to wait for 1–2 min after loading individual worms to ensure that the neurons reach the baseline of activity before recording the neuronal activity. Rarely, interneurons show spontaneous responses before delivering the stimulus. In this case, the worm was discarded. For data analysis, fluorescence intensities for each frame were extracted using customized neuron‐tracking MATLAB scripts.^39^, ^40^ In strains where both GCaMP and RFP were expressed (ZM9059 strain in this study), the ratio between intensity values were computed $\left(R\;=\frac{I_{\text{G}\_\text{ROI}}}{I_{\text{R}\_\text{ROI}}}\right)$ in order to minimize movement artifacts. When only GCaMP was available (CX10979, GT243, AQ4298, and AQ4300 strains in this study), fluorescence values were computed by subtracting background intensity ($F\;=\;I_{\text{G}\_\text{ROI}}-I_{{\text{G}}_{\text{Back}}})$. GCaMP and RFP intensities were measured as the mean pixel intensity of the 100 brightest pixels of a circular region of interest (ROI) of 10 pixel radius. Background intensities were subtracted to adjust for variations in lighting conditions and were measured as the mean pixel intensity of an ROI in a background region. Calcium traces were computed as the change in *R* or *F* from the baseline value $\left(\frac{\Delta R}{R_{0}}=\frac{R-R_{0}}{R_{0}}\right)$ or $\left(\frac{\Delta F}{F_{0}}=\frac{F-F_{0}}{F_{0}}\right)$. Baseline values were computed as the mean *R* or *F* prior to stimulus delivery.

## Conflict of Interest

The authors declare no conflict of interest.

## Supporting information

Supporting Information

Supplemental Movie 1

Supplemental Movie 2
