# Supplementary material for: Multimodal Stimulation in a Microfluidic Device Facilitates Studies of Interneurons in Sensory Integration in C. elegans
Source: Small. 2020 Jan 31;16(10):1905852. doi: 10.1002/smll.201905852 (PMC7720847; doi:10.1002/smll.201905852)
Supplement: Supplementary file 1 — Supporting Information [file SMLL-16-1905852-s001.pdf]

Copyright WILEY-VCH Verlag GmbH & Co. KGaA, 69469 Weinheim, Germany, 2020.

Supporting Information

for *Small*, DOI: 10.1002/smll.201905852

Multimodal Stimulation in a Microfluidic Device Facilitates Studies of Interneurons in Sensory Integration in *C. elegans*

*Yongmin Cho, Sol Ah Lee, Yee Lian Chew, Kirby Broderick, William R. Schafer, and Hang Lu**

Copyright WILEY-VCH Verlag GmbH & Co. KGaA, 69469 Weinheim, Germany, 2020.

Supporting Information

Multimodal Stimulation in a Microfluidic Device Reveals a Role of PVC Interneurons in Sensory Integration in *C. elegans*

*Yongmin Cho, Sol Ah Lee, Yee Lian Chew, Kirby Broderick, William R. Schafer, and Hang Lu**

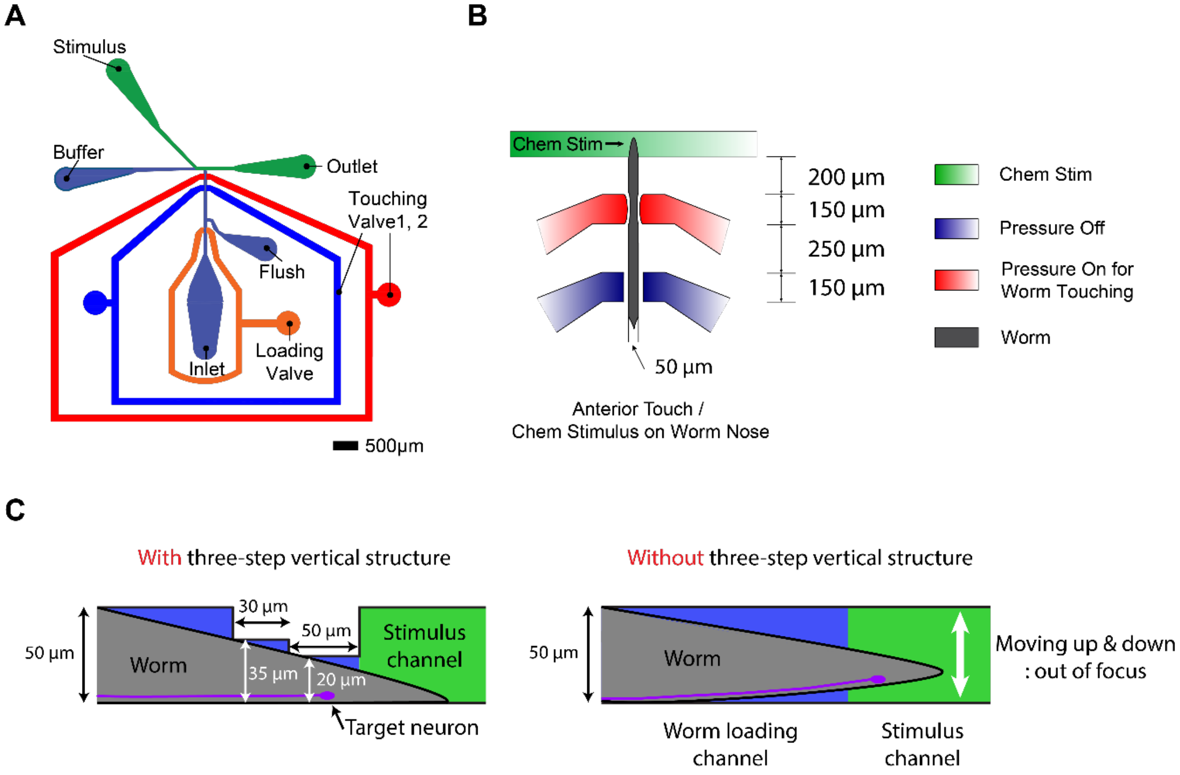

Figure S1. Overview of microfluidic device design and dimensions.

(A) The device is composed of a channel for worms (Loading arena, and Imaging and Flush channel), two sets of actuated membranes for mechanical stimuli (Touching valve 1, 2), one worm loading control valve (Worm loading valve), two inlets for chemical stimulus delivery (Buffer and Stimulus), and an outlet for chemical and worms. (B) Example moment of experiment with mechanical stimulus on anterior region and chemical stimulus on nose. The width of both PDMS actuators is 150 µm, the distance between chemical stimulus channel to first actuator is 200 µm and first and second actuator is 250 µm. The size of imaging channel is 50 µm x 50 µm (width and height) for day 1-2 adult worms. (C) Schematic illustration of immobilization method using a three-step vertical imaging channel.

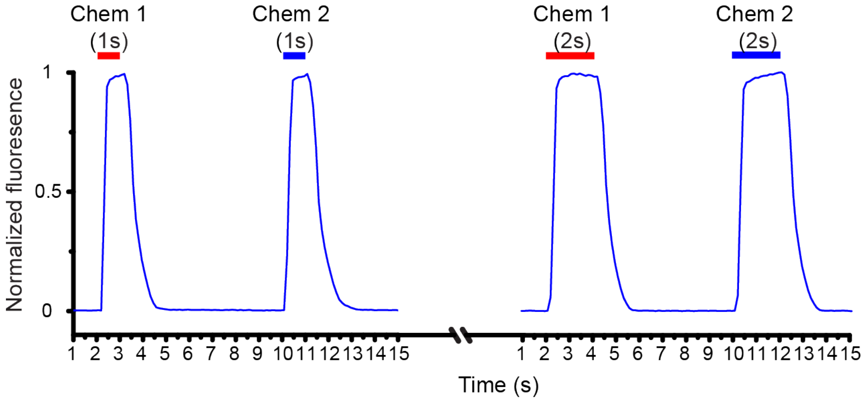

Figure S2. Using Y-shape connector (Nordson Medical, USA), the platform can deliver two different types of chemical stimuli. Temporal dynamics of two chemical stimuli control in the platform using fluorescein dyes in each stimulus stream (1s and 2s duration).

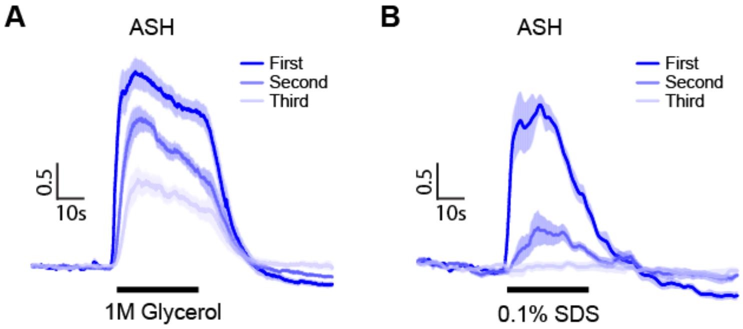

Figure S3. ASH responses to repeated chemical stimulation.
(A) 1M Glycerol (First: n = 13, Second: n = 10, and Third: n = 6) and (B) 0.1% SDS (First: n = 11, Second: n = 9, and Third: n = 6).

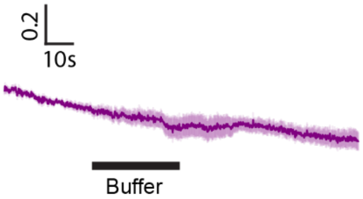

Figure S4. No clear PVC responses to buffer-to-buffer changes (n = 10).

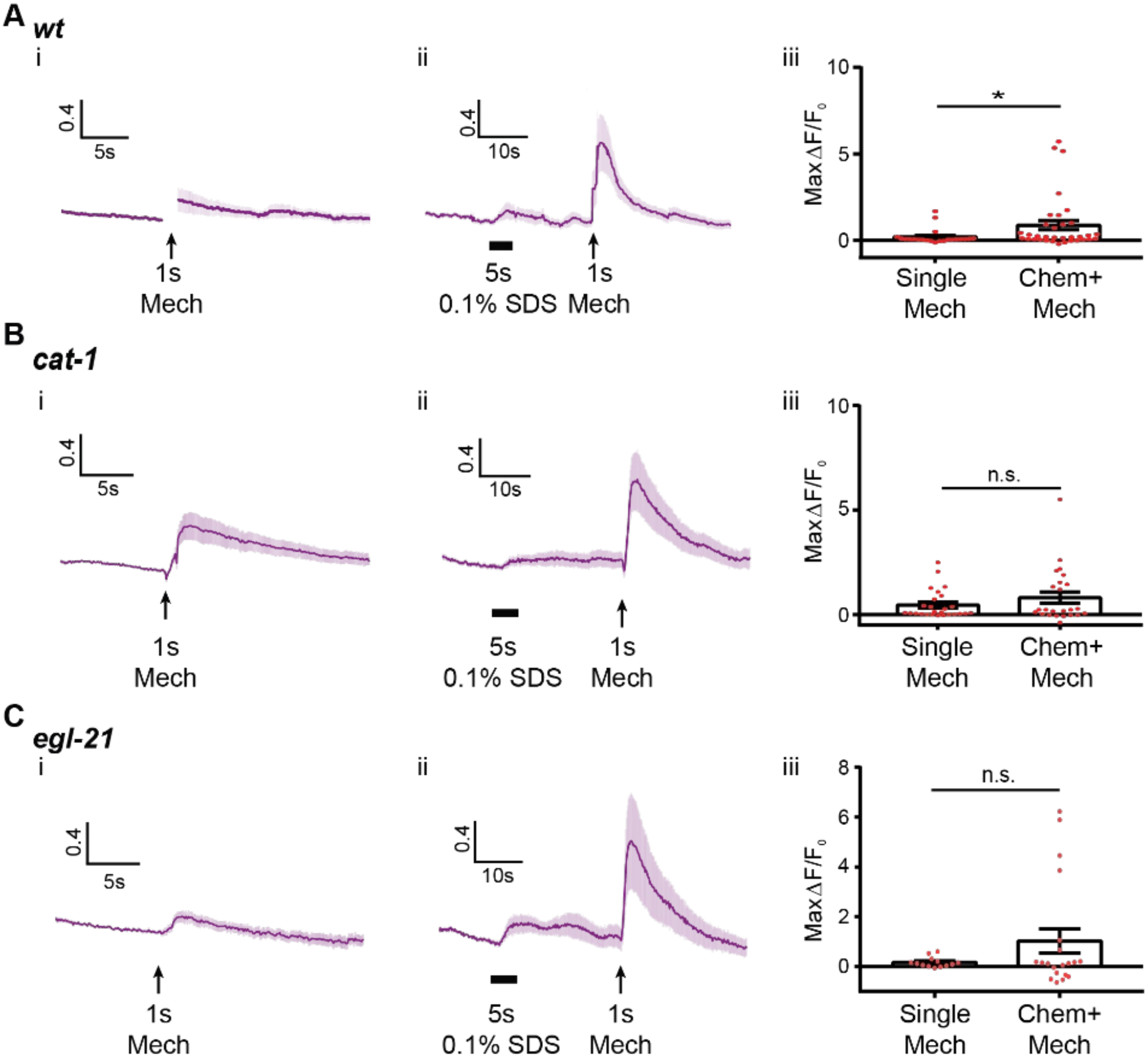

Figure S5. Activity of PVC interneurons responding to mechanical stimuli can be modulated by prior chemical stimulus. Monoamines and neuropeptides are likely to play a role in the multisensory integration in PVC. (A-C) Wild-type, *cat-1* and *egl-21* mutants were tested for PVC multisensory integration in conditions of i) single 1 s weak mechanical stimulus on posterior region, ii) 5 s 0.1% SDS chemical stimulus on tail and then 1 s weak mechanical stimulus on posterior region, and iii) quantitative comparison using the maximum responses of calcium transients (Mann-Whitney Test, * $p<0.05$, *** $p<0.001$). Data points represent maximum responses from individual trials. A) wild-type (single mech: n = 22 and chem + mech: n = 34), B) *cat-1* (*e1111*) mutant (single mech: n = 26 and chem + mech: n = 25), and C) *egl-21* (*n476*) mutant (single mech: n = 13 and chem + mech: n = 20). Error bars represent SEM. Single stimulus experiments are the same as in Figure 3E.

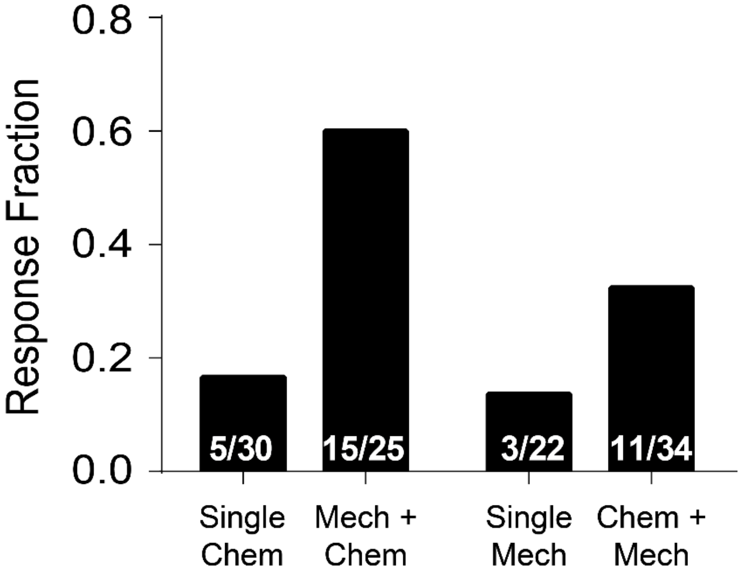

Figure S6. Response fraction of wildtype. Quantified the number of worms responding to stimulation depending on whether the calcium transient is above or below 0.5. In multimodal stimulation condition, the traces for second stimulation are used for this calculation. Numbers indicate the number of responding worms out of total trials.

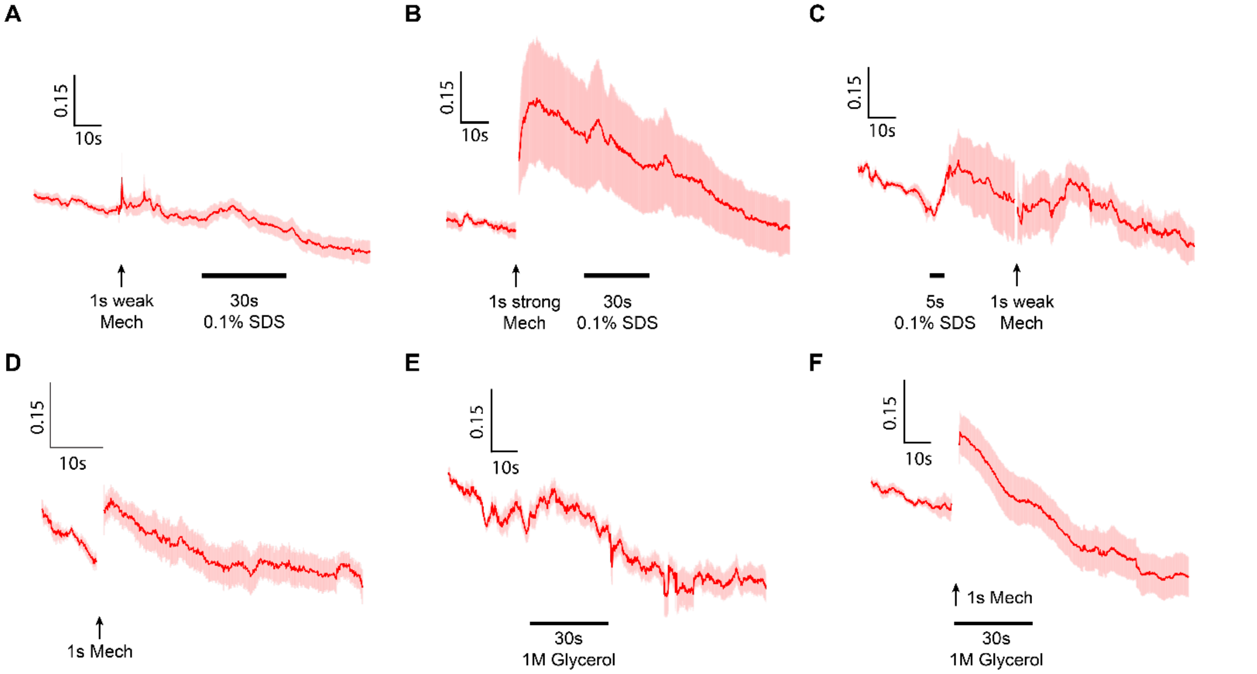

Figure S7. AVA responses to combinations of mechanical and chemical stimuli.
AVA responses to (A) 1s weak mechanical stimuli at 20 psi and then 30s 0.1% SDS stimuli (n = 42), (B) 1 s strong mechanical stimuli at 45 psi and then 30 s 0.1% SDS stimuli (n = 46), and (C) 5 s 0.1% SDS stimuli and then 1 s weak mechanical stimuli (n = 15).
AVA responses to (D) 1s mechanical stimuli at 20psi (n = 14) and (E) 30s 1M glycerol stimuli (n = 16). (F) AVA responses when both 1s mechanical and 30s 1M glycerol stimuli started at the same time (n = 24). Error bars represent SEM.
Two strains were used in this experiment: (A-C) ZM9059 (GCaMP6) and (D-F) QW625 (GCaMP3).

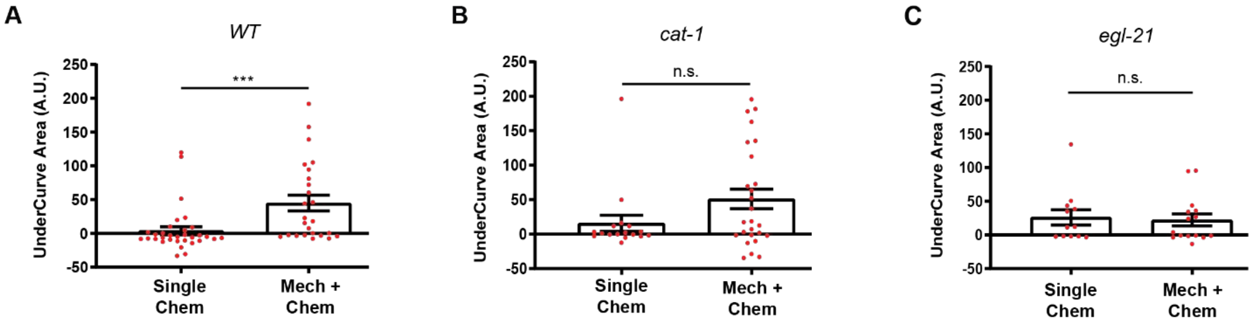

Figure S8. Quantitative comparison using area-under-the-curve of calcium transients in (A) wildtype and (B) *cat-1* and (C) *egl-21* mutants. (Mann-Whitney Test, *** $p<0.001$).

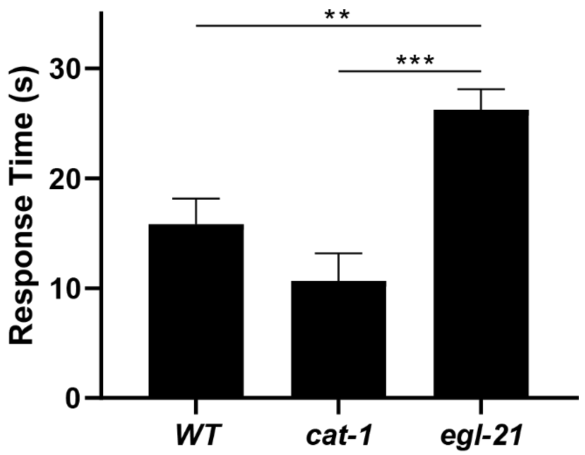

Figure S9. PVC neuronal response time to chemical stimulus upon prior mechanical stimulus. The time (s) between the start of stimulus application and the observation of 0.5 $\Delta F/F0$ values in calcium traces was quantified using calcium traces from Figure 5A ii (WT), 5B ii (*cat-1*), and 5 C ii (*egl-21*). Error bars are SEM. (Mann-Whitney Test, ** p< 0.01, *** p<0.001).

Movie S1. Calcium dynamics of the PVC command interneuron to 1 s mechanical stimulus on the posterior region and then 30 s 0.1% SDS stimulus to the tail. The transgenic animal shown here expresses GCaMP6 in PVC interneuron. White box indicates location of PVC neuron. Right graph shows the quantitative calcium trace and red circle indicates the current time point of video. Stimuli occur at vertical dash lines. 5x playback.

Movie S2. Calcium dynamics of PVC command interneuron to 5 s 0.1% SDS stimulus and then 1 s mechanical stimulus. 5x playback.
